# Exploring the Impact of Age-Related COVID-19 Messaging on Internalized Ageism in Older Adulthood

**DOI:** 10.1093/geroni/igaa057.3460

**Published:** 2020-12-16

**Authors:** Ruheena Sangrar, Stephanie Chesser, Michelle Porter

**Affiliations:** University of Manitoba, Winnipeg, Manitoba, Canada

## Abstract

Public health messages during the COVID-19 pandemic have indicated a higher risk for older people and/or those who have multiple health conditions. Subsequent societal discourse, however, has at times arguably protested the full protection and treatment of older people from COVID-19, potentially contributing to internalized ageism. To date, how older people interpret age-related pandemic messaging and discourse has not been explored. This study examined older adults’ perspectives of age-related COVID-19 messaging and societal discourse, as well as their perceptions of vulnerability, using a social constructionism framework. Adults age 65 to 89 years participated in semi-structured interviews about their thoughts and experiences with ongoing pandemic-related public messaging. Preliminary analysis suggests that participant perspectives of COVID-19 messaging are situated along a continuum of concern associated with contracting the virus. While some, for example, describe minimal concern, others express being fearful. Individual perceptions of safety appear to be informed, in part, by the presence or absence of an underlying health condition. Individual approaches to media criticism and consumption, personal risk-taking thresholds, financial stability, and social connectedness also appear to influence how the participants perceive pandemic-related messaging. Findings suggest the framing of COVID-19 and pandemic protocols, as well as the media’s sensationalization of age-related issues, can impact older peoples’ perceived vulnerability of contracting the virus. Future research is needed to understand the long-term implications of ongoing pandemic-related messaging on older adults’ experiences of aging, as well as the consequences such messaging could pose to for their health and social behaviors.

